# Supplementary material for: Targeted Delivery of Immunostimulatory CpG Oligodeoxynucleotides to Antigen-Presenting Cells in Draining Lymph Nodes by Stearic Acid Modification and Nanostructurization
Source: Int J Mol Sci. 2022 Jan 25;23(3):1350. doi: 10.3390/ijms23031350 (PMC8836026; doi:10.3390/ijms23031350)
Supplement: Supplementary file 1 [file ijms-23-01350-s001.zip › ijms-1484019-supplementary-done.pdf]

International Journal of *Molecular Sciences*

Targeted Delivery of Immunostimulatory CpG Oligodeoxynucleotides to Antigen-Presenting Cells in Draining Lymph Nodes by Stearic Acid Modification and Nanostructurization

Makoto Nagaoka [1], Wenqing Liao [1], Kosuke Kusamori [1] and Makiya Nishikawa [1]

[1] Laboratory of Biopharmaceutics, Faculty of Pharmaceutical Sciences, Tokyo University of Science, 2641 Yamazaki, Noda, Chiba 278-8510, Japan.

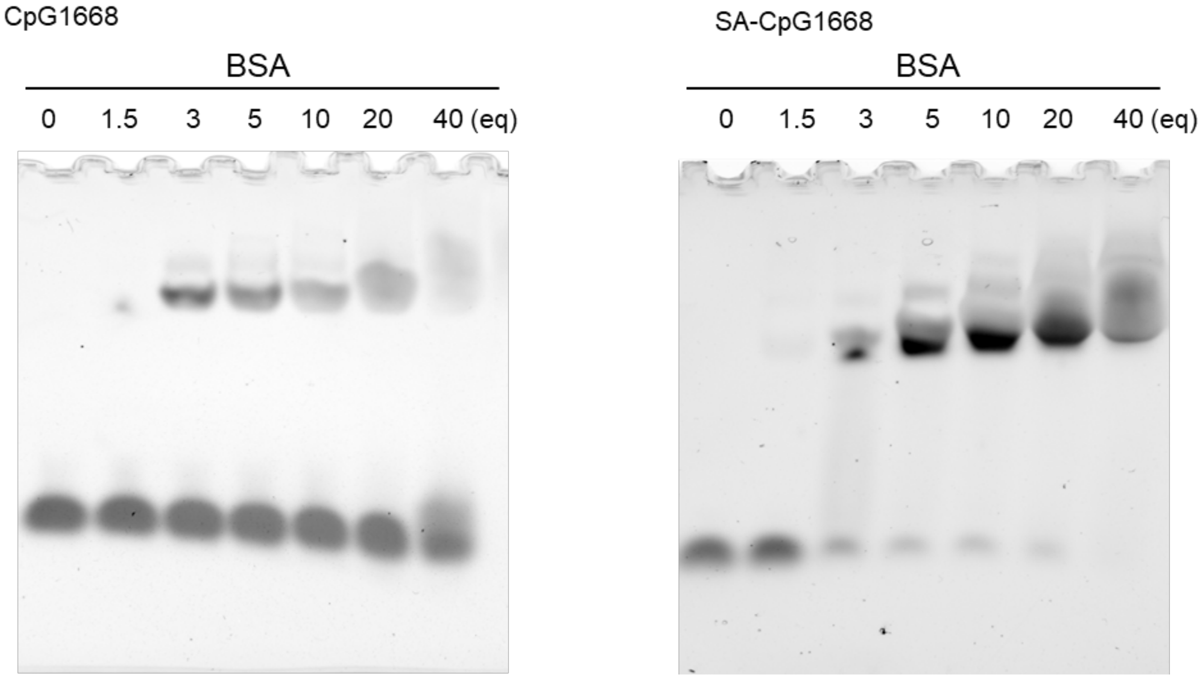

Supplementary Figure S1. Overall images of the gels shown in Figure 2. The upper and lower bands represent the bovine serum albumin (BSA) and DNA samples, respectively.

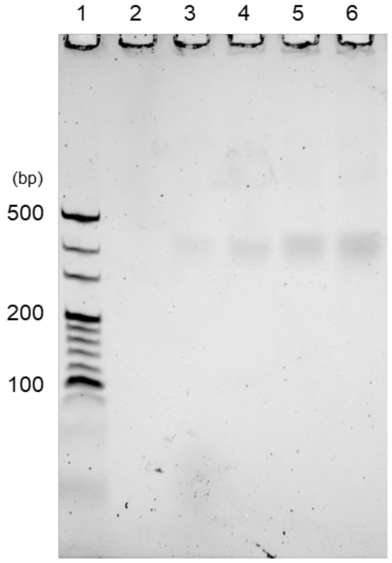

Supplementary Figure S2. PAGE analysis of DNA-free mouse plasma. Mouse plasma was run on a 6% polyacrylamide gel at 200 V for 30 min at 4 °C. Lane 1, 20 bp DNA ladder; lane 2, no plasma; lane 3, 20% plasma; lane 4, 40% plasma; lane 5, 60% plasma; and lane 6, 80% plasma.

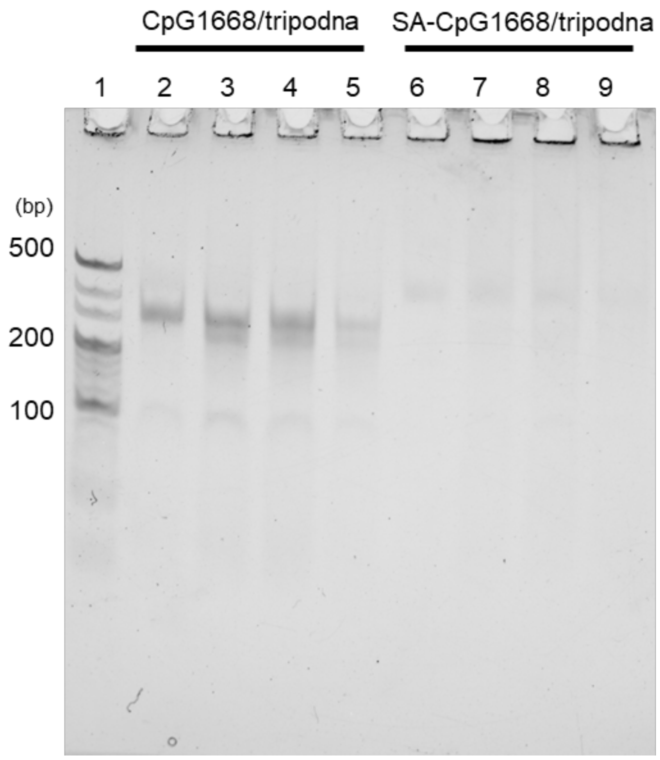

Supplementary Figure S3. Stability of CpG1668/tripodna and SA-CpG1668/tripodna in 10% plasma. Lane 1, 20 bp DNA ladder; lanes 2, 6, 0 h-incubation; lanes 3, 7, 1 h-incubation; lanes 4, 8, 2 h-incubation; lanes 5, 8 4 h-incubation.

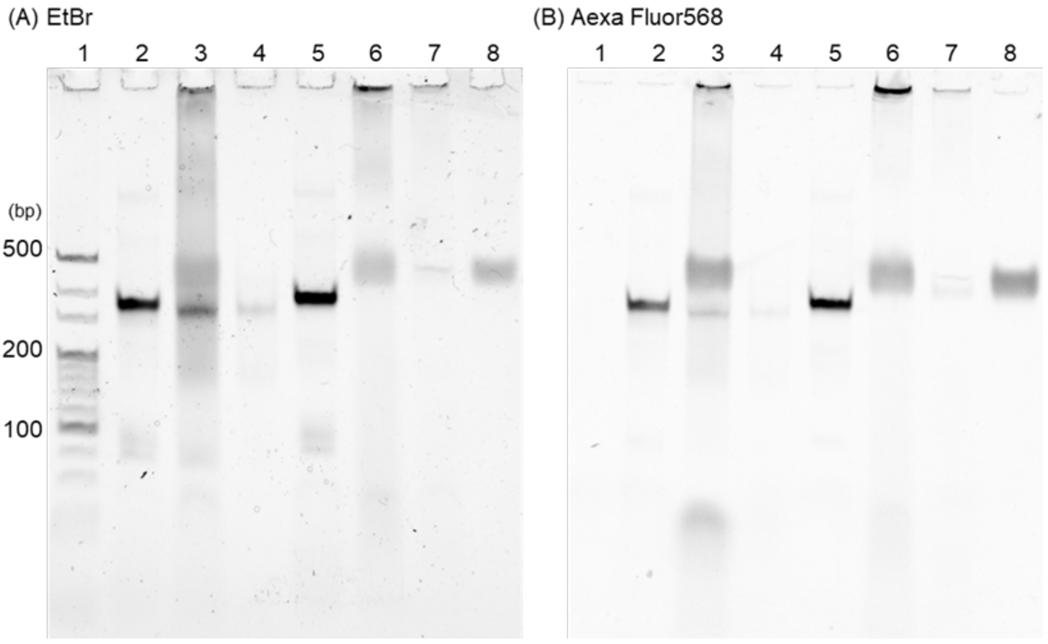

Supplementary Figure S4. PAGE analysis of the binding of SA-CpG1668/tripodna to plasma proteins in plasma after intravenous injection in mice. Mice were injected with CpG1668/Alexa Fluor568-labeled tripodna or SA-CpG1668/Alexa Fluor568-labeled tripodna at a dose of 10 nmol CpG/mouse into the tail vain. At 3 min after injection, plasma was collected and DNA in the plasma was detected by (A) EtBr or (B) the fluorescence of Alexa Fluor568. Lane 1, 20 bp DNA ladder; lane 2, CpG1668/tripodna (200 ng/well); lane 3, plasma of mice injected with CpG1668/tripodna; lane 4, plasma of mice injected with CpG1668/tripodna (10 times dilution); lane 5, SA-CpG1668/tripodna (200 ng/well); lane 6, plasma of mice injected with SA-CpG1668/tripodna; lane 7, plasma of mice injected with SA-CpG1668/tripodna (10 times dilution); and Lane 8, 80% plasma (no DNA).

^{1}H-NMR

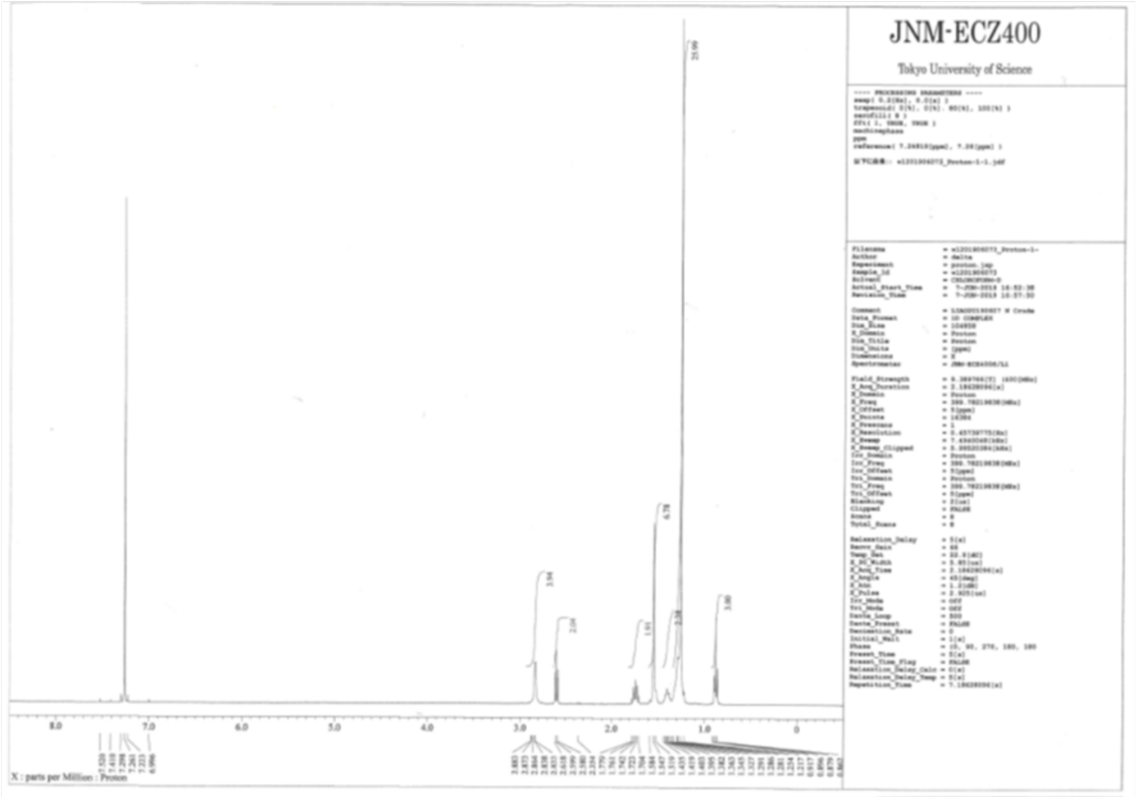

Supplementary Figure S5. ^{1}H-NMR (400 MHz, $CHCl_3$) spectrum of SA-NHS. Proton and carbon NMR spectra (^{1}H-NMR) were recorded on a JEOL JNM-ECZ400 (1H at 399.78 MHz) spectrometer (JEOL Ltd., Tokyo, Japan), with solvent resonance as the internal standard (^{1}H-NMR, $CHCl_3$ at 7.26 ppm). 1H-NMR (400 MHz, $CDCl_3$): 2.88-2.83 (m, 4H, N-CO-CH_2), 2.62-2.58 (t, 2H, -CH_2-COO-), 1.78-1.70 (tt, 2H, -CH_2-CH_2-COO-), 1.44-1.33 (m, 2H, -CH_2-CH_2-CH_2-COO-), 1.29-1.22 (m, 26H, -CH_2-Groups), 0.90-0.86 (d, 3H, CH_3-).

MALDI-TOF MS

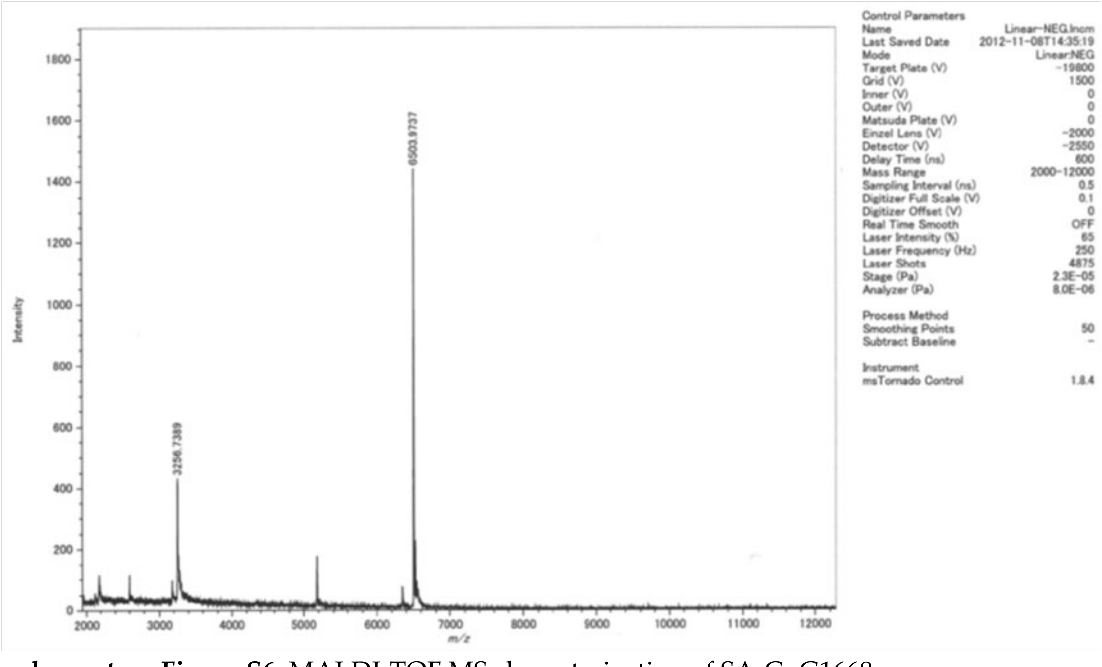

Supplementary Figure S6. MALDI-TOF MS characterization of SA-CpG1668.

Supplementary Table S1. Zeta potential of DNA sample.

Sample	Zeta potential (mv)
CpG1668	-7.3±0.9
SA-CpG1668	-9.6±0.4
CpG1668/tri-1	-12.7±1.4
SA-CpG1668/tri-1	-13.4±1.6
CpG1668/tripodna	-26.1±2.6
SA-CpG1668/tripodna	-25.2±1.8

The zeta potential of DNA samples was measured in 150 mM NaCl solution by using ELSZ-2000ZS (Otsuka Electronics Ltd., Osaka, Japan).
